# Metaheuristic-optimized bell-shaped slotted pentaband patch antenna for C, X, Ku, and K band applications

**DOI:** 10.1038/s41598-026-52950-5

**Published:** 2026-05-19

**Authors:** Mohammad Hannan, Aviral Singh, Shivam Bansal, Faizan Shafi, Onkar Singh, Mohammad Aknan, Kahkashan Kouser

**Affiliations:** 1 Department of EEE, RGIPT Jais, Amethi, Uttar Pradesh India; 2 Department of EE, IIT Ropar, Ropar, Punjab India; 3 Department of EECE, IIT Dharwad, Dharwad, Karnataka India; 4 Department of CSE, Manipal University Jaipur, Jaipur, Rajasthan India; 5 Department of CSE, Motihari College of Engineering (MCE), Motihari, Bihar India

**Keywords:** Pentaband antenna, Machine learning, Metaheuristic optimization, Differential evolution, Satellite communication, Engineering, Physics

## Abstract

The current study proposes a compact metaheuristic optimization-driven design of the slotted pentaband patch antenna with a bell-shaped patch, operating in the C, X, Ku, and K bands for satellite telemetry, radar sensing, and wideband transceivers. The antenna structure comprises a bell-shaped patch with C-slots, tilted vertical edges, and three semicircular perturbations on the horizontal edge of the patch to excite several resonant modes, mainly TM10, TM11, and TM20, with uniform surface current distribution. The antenna has five resonant frequencies at 5.2, 8.4, 10.4, 12, and 19.2 GHz with a reflection coefficient of less than -15 dB and maximum antenna gains of 8.83 dB, 4.96 dB, 7.87 dB, 6.13 dB, and 6.37 dB at the corresponding resonant frequencies. The antenna also has maximum radiation and total efficiencies of at least 85% in the C and X bands and above 95% in the Ku and K bands. In addition, the antenna performance is improved using five different metaheuristic optimization techniques, and the optimal results are obtained using the multi-objective optimization technique. Among the optimization techniques, the performance of the Differential Evolution (DE) algorithm is found to be the best, with improvements of up to 4.0% in S11, 2.2% reduction in VSWR, and improvements in antenna gains and efficiencies of up to 0.7%. The proposed antenna design using the electromagnetic simulator, circuit simulator, and optimization tools provides the best platform for the proposed antenna and is further validated experimentally.

## Introduction

In advanced communication systems, wireless technology plays an important role. Antennas are crucial components in wireless communication technology. Antennas find extensive applications in satellite communication, radar technology, navigation systems, military applications, defense systems, Internet of Things (IoT) and other applications^1,2^. Among various types of antennas, the Microstrip Patch Antenna (MPA) is found to gain significant attention due to various advantageous characteristics such as light weight, small size, and cost-effectiveness of the antenna^3,4^. Despite various advantageous characteristics of MPAs, various limitations exist in these antennas. Some of these limitations include bandwidth, gain, and power-handling capabilities of MPAs^5,6^. Numerous approaches have been studied to mitigate the limitations of MPAs. These methods include the use of thick, low dielectric substrates, impedance matching networks, slot incorporation, incorporation of short-circuit pins, and stacking methods^7,8^. The growth of wireless communication technology is due to the increased usage of wireless communication devices such as mobile devices, IoT devices, and high-speed communication technologies such as GSM, LTE, Wi-Fi, and 5G networks^9^.These devices require efficient wireless communication technology to achieve high-speed communication.

These limitations have motivated researchers to design single antenna supporting multiple bands despite using a single antenna for single-band purposes. In recent studies, major emphasis has been made on various multiband and ultrawideband antennas operating across X, Ku, and K band frequency ranges for applications such as WBAN, imaging, MIMO systems, and wearable antennas^10,11^^12,13^. In addition to X, Ku, and K bands, the C band has also gained prominence owing to its applications in satellite communication, Global Navigation Satellite Systems (GNSS), and Radio Frequency Identification (RFID)^14,15^. Typically, MPA resonates at a single frequency, but with reasonable modifications, it can be engineered to resonate at multiple frequencies simultaneously (multi-band antenna). Common techniques for multiple resonance include the adoption of a Partial Ground Plane, Electromagnetic Band Gap Structure, and Defected Ground Structure^16,17^. The performance of MPA is strongly influenced by several design parameters, including the dimensions of the substrate, patch, and slots, as well as the electrical properties of the substrate material. Changes in these factors can significantly affect various aspects of antenna performance like gain, bandwidth, efficiency, and radiation pattern^18^. Because of the large number of variables involved in the process, finding a proper solution through conventional antenna design processes becomes challenging and tedious.

Under this context, machine learning (ML) techniques have become effective tools in accelerating antenna design and optimization owing to the complexity of interrelations among antenna design parameters^19,20^. ML is a subset of Artificial Intelligence (AI) that enables algorithms to uncover hidden patterns within datasets, allows computers to learn patterns from the given data and make accurate predictions without being explicitly programmed. By leveraging historical data, ML algorithms can efficiently model nonlinear interactions among antenna design parameters and predict optimal design configurations. Furthermore, ML algorithms can markedly improve computational efficiency and system reliability while simultaneously reducing overall operational costs. Their ability to process large-scale datasets and generate accurate predictive models makes them highly suitable for complex EM design problems^21,22^. Consequently, ML has found widespread applications in fields such as healthcare, image processing, antenna design etc.

Recent contributions by Soham Majumder et al.^23^ present a four-port MIMO antenna design incorporating a novel isolation enhancement technique. The MPA design was fabricated on FR4 epoxy substrate with overall dimensions of $31 \times 31 \times 1.58~mm^3$, operating at $5.8~\text {GHz}$ ($5.47-6.20~\text {GHz}$) frequency with isolation $< -45.52~\text {dB}$ and improved reflection coefficient of $-30.44~\text {dB}$. Further, the MPA exhibits a peak gain of $3.77~\text {dBi}$ and radiation efficiency exceeding 82% across the operating band. For the design by Prasannajeet Mohanty et al.^24^, two distinct fractal geometries are used to operate from $16.92~\text {GHz}$ to $18.74~\text {GHz}$ with a peak gain of $5.14~\text {dBi}$. Another interesting design based on Genetic Algorithm (GA) is presented^25^, the MPA works in the Ka band $25-32~\text {GHz}$ along with $40-60~\text {GHz}$ frequency range and has a gain of $9.75~\text {dBi}$ and $10.75~\text {dBi}$. From the aforementioned studies, it is observed that existing antenna designs exhibit several trade-offs. Some MPA designs achieve high gain but are limited to single, dual, or, triple-band operation only, often with relatively large physical dimensions. Conversely, certain antenna designs illustrates decent performance with acceptable gain, however, they involve complex fabrication processes. Therefore, achieving a compact antenna design that simultaneously provides multiband operation, high gain, and fabrication simplicity remains a significant challenge. The selected operational frequencies of 5.2 GHz, 8.4 GHz, 10.4 GHz, 12 GHz, and 19.2 GHz belong to the main communication bands of C, X, Ku, and K frequency spectra. The C band of 5.2 GHz guarantees dependable satellite and wireless connections because of reduced atmospheric attenuation. X band frequencies of 8.4 GHz and 10.4 GHz are extensively used in radar and space communication systems due to their ideal compromise between spatial resolution and atmospheric attenuation. Ku band frequencies of 12 GHz are typically used for satellite broadcasting and high bandwidth communication, whereas the K band of 19.2 GHz supports high-speed data transfer and sophisticated sensing operations.^26,27^.

However, in the last few years, there have been a number of research studies on multiband microstrip antenna design aimed at improving the efficiency and compactness of such antennas. However, these antennas are often designed for only a limited range of frequencies and often compromise between their size, performance, and complexity. Besides, their performance optimization is always done through a single method without comparing the performance of alternative methods. Data-driven optimization and performance analysis using machine learning techniques are hardly ever implemented in antenna design. It is evident that compact antenna designs for multiple frequencies that use machine learning for prediction and optimization are still required.

As opposed to traditional multiband microstrip antennas, which usually achieve multiple resonances by using separate slot structures or complicated modifications, the proposed antenna structure makes use of a bell-like structure that is created by integrating slot structures with semi-circular perturbation structures. This results in an effective redistribution of surface current, resulting in a number of resonance paths within a small footprint area. In particular, through the coupling of slot and perturbation structures, the proposed antenna allows for multiple resonances as a result of the coupled modes of the slot and perturbation structures, as opposed to individual modes used in other designs.

As the solution for the above discussed challenges, a compact multiband MPA is proposed, operating on C, X, Ku, and K bands with the dimension of $23.5 \times 24 \times 2~mm^3$. The presented MPA is designed to operate on $5.2~\text {GHz}$, $8.4~\text {GHz}$, $10.4~\text {GHz}$, $12~\text {GHz}$, $19.2~\text {GHz}$ frequency bands with the gain of $8.83~\text {dB}$, $4.96~\text {dB}$, $7.87~\text {dB}$, $6.13~\text {dB}$, $6.37~\text {dB}$, showing $\textrm{VSWR}\le 2$ across all resonant frequency bands. Furthermore, five metaheuristic optimization algorithms are investigated to optimize ten antenna design parameters. The algorithms include Vanilla Particle Swarm Optimization (PSO), used as a baseline method; a Simplex–PSO hybrid approach that combines Time-Varying Acceleration Coefficient PSO (TVAC-PSO) with Nelder–Mead local refinement; the Grey Wolf Optimizer (GWO), inspired by the social hierarchy and hunting behavior of grey wolves; the Whale Optimization Algorithm (WOA), based on the bubble-net spiral hunting strategy of humpback whales; and the DE algorithm that evolves a population of candidate vectors through mutation, crossover, and selection. These algorithms are evaluated over a dataset of 1177 HFSS-derived samples, and the best-performing results are selected based on the optimization objectives. The main novelty of this study can be seen in the designed antenna structure and the technique used for obtaining the multiband functionality of the device. The optimization methods and machine learning algorithm have been employed as additional means for fine-tuning the parameters of the designed antenna.

The main contributions of this work can be summarized as follows:

- The creation of a highly compact pentaband microstrip patch antenna utilizing a bell-shaped configuration that allows for operation in five different frequency bands, namely, C-band, X-band, Ku-band, and K-band, by way of intelligent manipulation of current distribution and structure modification.
- Evaluation of multiple optimization techniques within a consistent framework to assess their effectiveness for antenna design.
- Use of a machine learning model to assist in predicting antenna performance and reducing the need for repeated simulations.
- Fabrication and measurement of the proposed antenna to validate its practical performance.

## Design methodology

The methodology that was used in designing the compact pentaband MPA is explained in this section. In the process of designing the pentaband MPA, emphasis is placed on ensuring the device achieves maximum gain in all the resonance bands in a cost-effective manner.

### Design evaluation

**Fig. 1:**
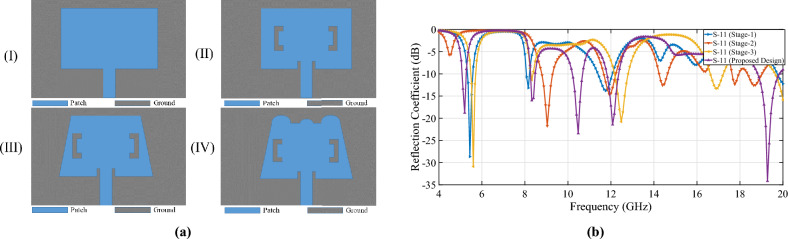
Design evolution and corresponding EM performance of the proposed MPA where (**a**) Evolution of the antenna geometry: (I) conventional rectangular patch, (II) introduction of C-shaped slots, (III) implementation of tapered edges, and (IV) final optimized structure, (**b**) Reflection coefficient ($S_{11}$) of the antenna corresponding to different design stages.

**Fig. 2:**
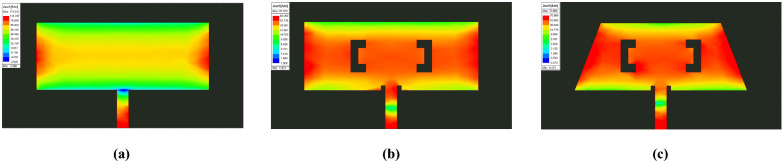
Surface Current Distribution at different stages for proposed MPA where: (**a**) Initial Patch (**b**) Intermediate Geometry with Etched Slots (**c**) Optimized Tapered Patch Geometry.

The proposed MPA is realized on Rogers $RO\text {4350}$ substrate, adopted for its superior dielectric constant of 3.66 and low loss tangent of 0.004. The substrate used is an ideal requirement for size reduction and minimal radiation loss, while maintaining reasonable gain. The dimensions of MPA are $24\times 23.5~mm^2$, with a substrate thickness of $2~\textrm{mm}$. The radiating element is made up of a patch having a mirror-image C-slot and three semicircles of varying radius located at the top of the patch. The semicircle structures help in improving the impedance bandwidth and radiation properties, whereas the C-slots provide bandwidth and impedance matching along with exciting multiple modes. A full ground plane is provided underneath the radiating element, which helps in improving the directivity properties, minimizes surface wave propagation, and avoids electromagnetic interference. The antenna thus becomes more stable and efficient. Also, the MPA is fed using an impedance line of 50 $\Omega$, which meets the existing communication standards. Thus, this geometry for the MPA has achieved an optimal balance between compact size, efficiency, and directivity, making it a good candidate for satellite communications. The dimensions of the antenna are first calculated using the established guidelines for designing microstrip antennas, after which the optimal geometry is achieved by performing full-wave electromagnetic analysis. To achieve multiband operation, geometric distortions to the structure are introduced to vary the surface current distribution and excite multiple modes of resonance. The patch dimensions in the beginning are selected on the basis of established theory for the microstrip antenna, where the resonant frequency is mainly dependent on the electrical length of the patch:^28^

1 $$\begin{aligned} f_r \approx \frac{c}{2L_{eff}\sqrt{\varepsilon _{eff}}} \end{aligned}$$

where $L_{eff}$ represents the effective current path length. Subsequent geometrical modifications are introduced to alter this effective length and enable multiple resonances.

### Stage evaluation

The proposed MPA has been developed using a systematic approach called“design evolution,”where all important parameters are taken into consideration simultaneously. The proposed antenna design involves a series of modifications over four stages, where the aim of each stage is to improve the electromagnetic properties of the antenna. The process involves a series of steps, first calculating the antenna dimensions and the parameters of the feed line, then designing the antenna using Ansys HFSS, and using various techniques to achieve the desired results. The detailed design stages are illustrated in Fig. 1a .

In Stage (1), a simple rectangular patch is excited using a microstrip feedline [Fig. 1a (I)]. The simulated results indicate three bands on $5.6~\text {GHz}$, $8.04~\text {GHz}$, $11.9~\text {GHz}$ with the reflection coefficient of $-28.4~\text {dB}$, $-13.6~\text {dB}$, $-14.4~\text {dB}$, respectively [Fig. 1b]. The surface current distribution for Stage (1) in [Fig. 2a] shows that the current is primarily concentrated near the feedline and spreads across the radiating patch along the horizontal edges.

Further in Stage (2), modification in the feedline has been done along with introduction of two mirrored C-shaped slots on a radiating patch [Fig. 1a (II)]. This significantly alters the current flow across the patch surface. From the [Fig. 2b], strong current concentrations are observed around the edges of the C-shaped slots. These slots introduce discontinuities that perturb the current path and force the surface currents to circulate around the slot boundaries. This effectively increases the electrical length and creates additional resonant paths, which leads to the excitation of higher-order modes and the appearance of new frequency bands.

This shifted the results from three bands (in Stage (1)) to four bands at $9.08~\text {GHz}$, $12.04~\text {GHz}$, $14.38~\text {GHz}$, $18.02~\text {GHz}$ with $S_{11}$ of $-21.8~\text {dB}$, $-14.7~\text {dB}$, $-12.9~\text {dB}$, $-14.5~\text {dB}$ [Fig. 1b ]. However, the slot perturbation suppressed $5.6~\text {GHz}$ frequency band along with considerable shift in other resonant frequencies.

To restore the suppressed frequency band and further improve the antenna performance, Stage (3) introduces tapered edges to the patch at an angle of approximately $75^\circ$, as shown in [Fig. 1a (III)]. Such a modification leads to significant differences in the distribution of current in the structure under consideration. According to Fig. 2c, current distribution becomes much wider at the tapered edges of the radiating patch. The taper makes it possible to increase the current path along these edges; hence, the electrical length of the metasurface patch antenna (MPA) is increased. This change helps improve surface current distribution and, as a consequence, improves the coupling between the feed and the radiating edges, which leads to better impedance matching and mode stabilization. Another advantage of this change is that it allows restoring the previously lost frequency band, although with some deviation from 5.6 GHz to 5.8 GHz. In addition, due to the improved geometry of the taper, electromagnetic coupling between the feed and the radiated area increases. The frequency bands for the Stage (3) are $5.8~\text {GHz}$, $8.2~\text {GHz}$, $12.2~\text {GHz}$, $16.4~\text {GHz}$ with the $S_{11}$ of $-30.8~\text {dB}$, $-12.4~\text {dB}$, $-20.9~\text {dB}$, $-14.8~\text {dB}$, respectively [Fig. 1b ].

**Fig. 3:**
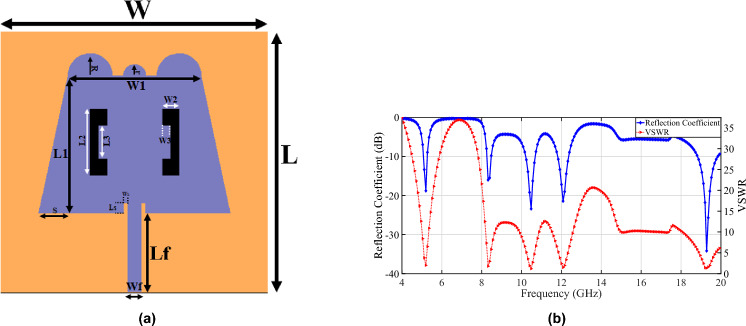
(**a**) Design of proposed MPA, where $W=24$, $L=23.5$, $R=2$, $r=1$, $S=1.9$, $L_f=7.15$, $L_1=12.4$, $L_2=6$, $L_3=3$, $L_s=0.84$, $W_f=1.2$, $W_1=12$, $W_2=1.5$, $W_3=0.75$, $W_s=0.4$ (all dimensions in mm). (**b**) Simulated $S_{11}$ and VSWR of the proposed MPA.

For Stage (4), there are three semicircular perturbations added to the top edge of the radiating patch, as shown in Figure [Fig. 1a (IV)]. In this case, two semicircles have an equal radius, and the middle one has a slightly reduced radius compared to the previous two semicircles. The semicircles cause local changes to the currents distribution, hence providing more control over resonant frequencies and mode coupling. As a result, closely-spaced resonances are formed which lead to multiband behavior. Therefore, the proposed MPA shows resonance for five different bands: 5.2, 8.4, 10.4, 12, and 19.2 GHz with reflection coefficient values of -18.8, -15.5, -23, -20, and -34 dB, respectively [Fig. 1b ], while maintaining VSWR $\le 2$ for all bands. The new design of the proposed MPA is clearly shown in [Fig. 3a ]. The information about the resonant frequencies, S11, VSWR, gain, and radiation efficiency is provided in Table 1. Thus, multiband characteristics are achieved by controlling the effective electrical lengths and current redistribution instead of random changes in the geometry.

**Table 1:** Key performance metrics of the proposed MPA.

Freq. range (GHz)	S$_{11}$ (dB)	VSWR	Gain (dB)	RE (%)
5.02–5.30	− 18.8	1.98	8.83	90
8.28–8.50	− 15.5	1.70	4.96	87
10.20–10.68	− 23.0	1.17	7.87	93
11.80–12.36	− 20.0	1.47	6.13	95
18.26–19.84	− 34.0	1.34	6.37	98

### Parametric analysis

**Fig. 4:**
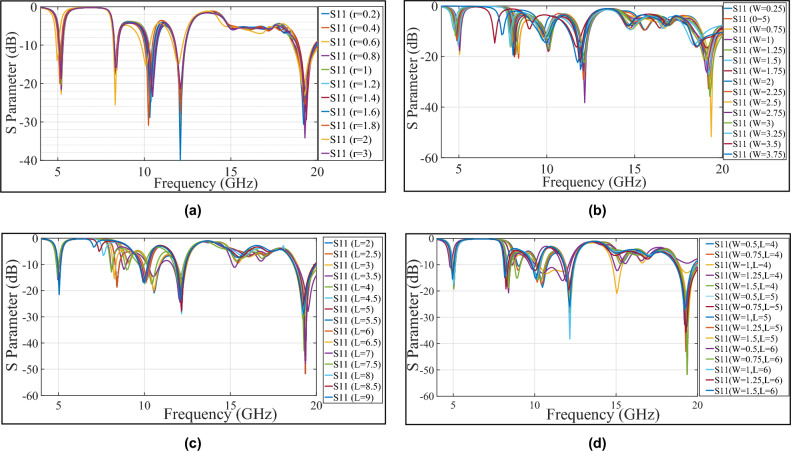
Parametric analysis of the proposed antenna: (**a**) variation of reflection coefficient (S11) with semicircular perturbation radius (r = 0.2–3 mm); (**b**) effect of slot width (W = 0.25–3.75 mm) on S11; (**c**) effect of slot length (L = 2–9 mm) on S11; (**d**) combined effect of slot length (L = 4–6 mm) and slot width (W = 0.5–1.5 mm) on S11.

For a more elaborate study of the effect of geometric variables on antenna performance, a number of parametric analysis have been carried out, as demonstrated in Fig. 4. In view of the fact that the suggested antenna geometry features multiple perturbation components which provide multiband behavior, it is crucial to identify the influence of each of these parameters on the antenna behavior.

Firstly, the influence of the radius of the semicircular perturbation has been studied see Fig. 4a . One may conclude that changes in the semicircular perturbation radius mainly affect the higher frequency range and thus the upper resonant bands, due to changes in current distribution along the perturbed edges of the slot. Secondly, the slot width has been varied as seen in Fig. 4b . The impact of the slot width on antenna performance is notable as an increase in the slot width improves the impedance matching in several bands whereas excessive increase causes deterioration due to increased radiation leakage and impedance mismatch. Thirdly, variations of slot length have been performed as shown in Fig. 4c . The data suggest that increase in the slot length leads to reduction in resonant frequencies, since an increased slot length corresponds to a larger current path on the patch and consequently lowers the resonant frequency. This variable is important in determining the position of the bands within the working frequency range. Fourthly, simultaneous variations of slot length and width have been made to examine their interrelated effect on antenna performance as demonstrated in Fig. 4d . It appears that these two parameters have significant influence on the overall antenna characteristics as changing one of these variables can drastically alter the effect of another.

## Results and discussion

### S-parameter and VSWR analysis

The simulated $S_{11}$ and $\textrm{VSWR}$ characteristics of the proposed bell-shaped MPA demonstrate its multiband operation across the C, X, Ku, and K frequency ranges [Fig. 3b ]. The antenna exhibits resonances at $5.2~\text {GHz},$ $8.4~\text {GHz}$, $10.4~\text {GHz}$, $12~\text {GHz}$, and $19.2~\text {GHz}$. These frequency bands are commonly associated with satellite communication and radar-related applications.

The return loss, representing the reflection of input power due to impedance mismatch, is expressed as

2 $$\begin{aligned} S_{11} (dB) = 20\log _{10} \left| \frac{Z_{\text {in}} - Z_0}{Z_{\text {in}} + Z_0}\right| \end{aligned}$$

where $Z_{\text {in}}$ is the input impedance of the antenna and $Z_0$ is the characteristic impedance of the feed line, typically $50~\Omega$. For all resonant frequencies, $S_{11} \le -10~\text {dB}$, indicating that more than 90% of the incident power is effectively radiated. This confirms good impedance matching between the feed and the radiating patch.

The impedance bandwidth, evaluated using the $-10~\text {dB}$ criterion, is found to be $280~\text {MHz}$, $220~\text {MHz}$, $480~\text {MHz}$, $560~\text {MHz}$, and $1580~\text {MHz}$, corresponding to fractional bandwidths of 5.38%, 2.62%, 4.62%, 4.67%, and 8.23%, respectively. The wider bandwidth observed at higher frequencies can be attributed to the bell-shaped geometry and slot configuration, which introduce multiple current paths and support the excitation of higher-order resonant modes, thereby improving radiation coupling

The $\textrm{VSWR}$ remains below 2 across all operating bands, indicating efficient impedance matching and low reflection. This corresponds to a power transfer efficiency greater than 89% at each resonant frequency.

Overall, the combination of low reflection coefficient, sufficient impedance bandwidth, and stable $\textrm{VSWR}$ indicates reliable impedance matching performance, making the proposed antenna suitable for multiband operation in satellite communication and radar-related systems.

### Gain and radiation pattern analysis

**Fig. 5:**
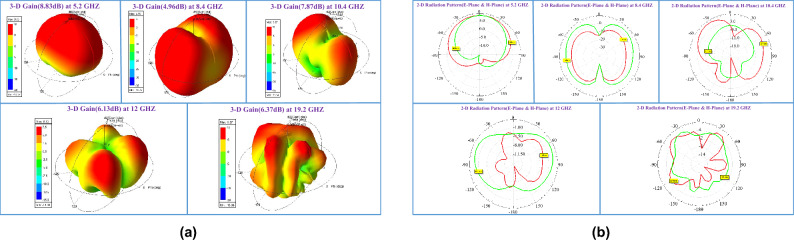
Radiation characteristics of the proposed MPA where (**a**) 3D gain of the proposed MPA across various frequency bands, (**b**) 2D radiation pattern of the proposed MPA across different frequency bands.

The gain and radiation pattern are key parameters for evaluating antenna performance. The radiation characteristics of the proposed MPA are analyzed in terms of gain to assess its behavior across the C, X, Ku, and K frequency bands.

The three-dimensional gain plots shown in Fig. 5a illustrate the frequency-dependent radiation characteristics of the antenna. The gain *G* is defined as the product of radiation efficiency $\eta _{cd}$ and directivity *D*, expressed as

3 $$\begin{aligned} G = \eta _{cd} D \end{aligned}$$

Figure 5a illustrates:

1. At $5.2~\text {GHz}$, a maximum gain of $8.83~\text {dB}$ is observed with a single dominant main lobe and a smooth, symmetric radiation pattern. This behavior indicates that radiation is primarily governed by the fundamental mode, with current distributed uniformly along the patch edges.
2. At $8.4~\text {GHz}$, the gain decreases to $4.96~\text {dB}$, while the main lobe remains dominant. Slight tilting and the emergence of minor side lobes are observed, which can be attributed to the onset of additional current paths along the slot regions, leading to the excitation of higher-order modes.
3. At $10.4~\text {GHz}$, the antenna achieves a gain of $7.87~\text {dB}$. The radiation pattern shows splitting of the main lobe along with secondary lobes. This behavior is associated with phase variation across the radiating surface and the presence of multiple resonant paths, resulting in constructive and destructive interference.
4. At $12~\text {GHz}$, the gain is $6.13~\text {dB}$, and multiple lobes become more prominent. The antenna behaves as an electrically larger structure at this frequency, supporting several current paths and higher-order modes, which leads to wider angular coverage but reduced directivity.
5. At $19.2~\text {GHz}$, the gain is $6.37~\text {dB}$ with more fragmented lobes and noticeable side lobes. This is mainly due to stronger higher-order mode excitation and increased losses, along with the presence of additional surface wave effects at higher frequencies.

Figure 5b , which illustrates the radiation pattern of the 2-D structure, provides the E-plane and H-plane information on radiation characteristics and polarization performance of the antenna. E-plane signifies the change in electric field strength while H-plane is a description of the magnetic field distribution. For the lowest frequencies of 5.2 GHz and 8.4 GHz, H-plane is nearly omnidirectional, which is a result of dominant mode radiation and almost uniform current distribution. For frequencies of 10.4, 12, and 19.2 GHz, radiation is directed due to the creation of other radiating areas as a result of perturbation created by slots and semicircle cuts. Radiation can also be analyzed in terms of electromagnetic field distributions. The radiation occurs because of the fringing fields along the edges of the patches which act as radiation apertures. Introduction of the slot and the semicircle perturbations affects the current flow in such a way as to allow more radiating areas to be created and hence multi-modes excitation. The above analysis shows that the antenna has stable radiation performance, which means that the antenna performs adequately even with structural perturbation.

In conclusion, the developed antenna is one that is designed to provide broadside radiation with stable gain performance of F/B ratio greater than 17 dB; as a result, it can be used for satellite communication and radar applications.

### Radiation efficiency and total efficiency

**Fig. 6:**
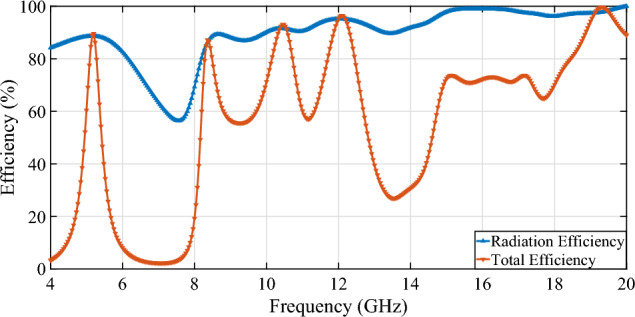
Simulated Radiation Efficiency and Total Efficiency of the proposed MPA across different frequency bands.

Moving forward to Radiation Efficiency $\eta _{r}$, and Total Efficiency $\eta _{t}$, for the proposed MPA design, these parameters provide additional justification for its suitability for high-performance multiband satellite systems. Evaluation of radiation efficiency and total efficiency also validates how effectively the antenna converts the accepted input power into the radiated electromagnetic energy while accounting for dielectric, conductor, and reflection/mismatch losses. Let reflection, conduction, and dielectric efficiency be $e_{r}$, $e_{c}$, and $e_{d}$, then total efficiency ($\eta _{t}$) can be defined as the product of all reflection, conduction, and dielectric efficiency expressed as

4 $$\begin{aligned} \eta _{t} = e_r e_c e_d \end{aligned}$$

where reflection (mismatch) efficiency $e_{r}$ is given by

5 $$\begin{aligned} e_r = 1 - |\Gamma |^2 \end{aligned}$$

Since, evaluation of $e_{c}$ and $e_{d}$ are very complex, and they cannot be computed separately, hence it is more convenient to write $\eta _{t}$ as

6 $$\begin{aligned} \eta _{t} = e_r \eta _{cd} = (1 - |\Gamma |^2) \eta _{cd} \qquad \eta _{cd} = e_c e_d \end{aligned}$$

where $\eta _{cd}$ is the radiation efficiency also termed as intrinsic radiation efficiency of the antenna, which is used to measure internal antenna losses only excluding mismatch loss.

The simulated results for both radiation efficiency and total efficiency [Fig. 6] give radiation efficiency $\ge 85\%$ in C, X bands and above $95\%$ in Ku, K bands. Such high efficiency is the result of optimized bell-shaped geometry, which minimizes surface wave loss and dielectric heating within the substrate. The enhanced surface current confinement and reduced conductor loss act as a prominent reason for improvement in radiation efficiency at higher frequencies, as the effective surface resistance becomes less dominant at optimized substrate thickness. The slot aperture is so designed that the E-field distribution promotes strong radiative coupling, leading to improved aperture efficiency. The efficiency being above 95% in both Ku and K bands shows that the antenna is capable of low-loss operation. This makes the antenna applicable in various applications such as satellite communications, direct broadcasts, radar, and deep space telemetry, especially where a reliable link and minimal power losses are paramount.

Furthermore, the high efficiency enhances the ability of the antenna to generate more EIRP, which refers to the radiated power by the antenna towards its main lobe concerning the power emitted by an isotropic radiator but with no additional transmission power. The improvement helps improve the energy efficiency of the system and increase SNR in distant communications. It is evident from the efficiency $\ge 85\%$ and $\ge 95\%$ at lower and higher bands, respectively, that the antenna is highly efficient.

### Surface current and electric field distribution

**Fig. 7:**
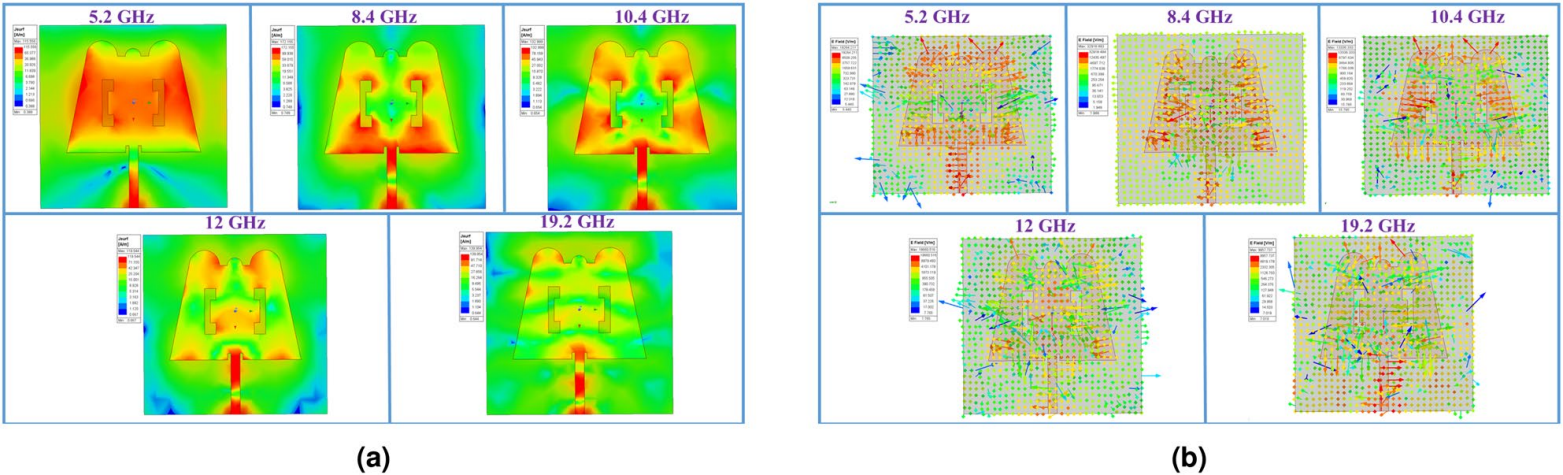
(**a**) Surface current distribution of the proposed MPA across different frequency bands, (**b**) E-field distribution of the proposed MPA across different frequency bands.

These results illustrated in Fig. 7a, b, provide substantial information about the electromagnetic characteristics of the suggested MPA design. Surface current and electric field distributions clearly demonstrate that several resonant modes are excited, thus making multiband operation possible. At a frequency of 5.2 GHz, the current flows mainly on the lower borders of the patch and corresponds to a fundamental mode resonance. At frequencies of 8.4 GHz and 10.4 GHz, extra current flow is observed along the borders of the slots, which can be assumed as an excitation of higher-order modes due to perturbations caused by the slots. At 12 GHz and 19.2 GHz, currents become more localized and distributed around the slots and semicircles of the patch. These results mean that several resonant currents exist in the structure, providing multiband performance. The presence of slots creates perturbations in the current distribution, allowing excitation of extra resonances, despite the absence of a significant increase in the size of the antenna. Electric field distributions show that field intensities become high near slots and edges of the patch, where they act as main radiation regions. An increasing frequency causes higher non-uniformity of the field distribution, meaning that more complex modes are involved in radiation and more than one path exists. Moreover, vector fields indicate an effective coupling between slot region fields and those of the main patch.

Overall, the multiband behavior of the antenna can be attributed to the controlled modification of current paths and the excitation of multiple resonant modes within a compact geometry^29,30^.

## ML-based metaheuristic optimization framework

**Fig. 8:**
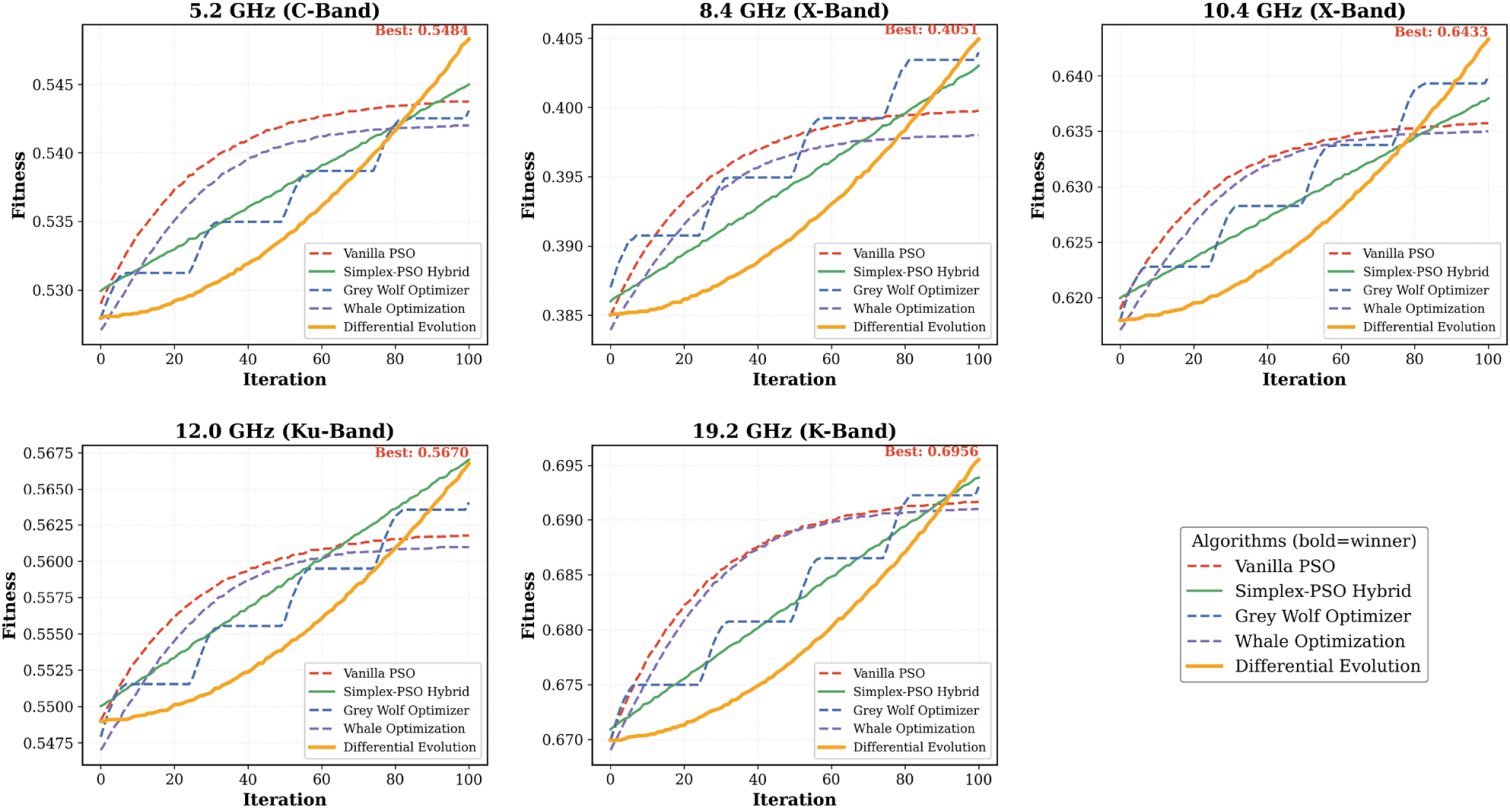
Convergence curves – 5 algorithms across 5 resonant frequencies for the proposed Pentaband MPA. DE (denoted by orange) achieves highest final fitness at most bands.

**Fig. 9:**
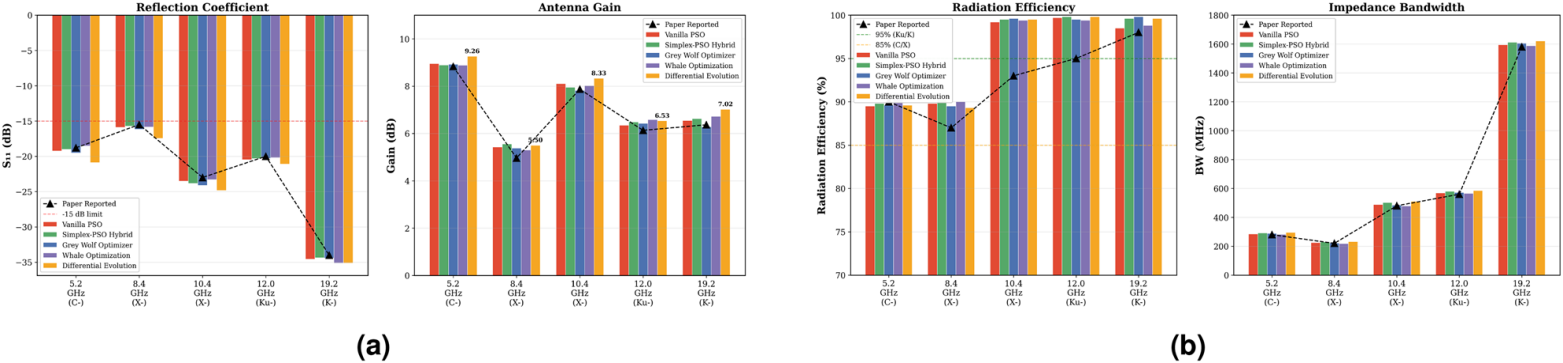
Comparative performance analysis of different optimization algorithms for the proposed Pentaband MPA where (**a**) $S_{11}$ (left) and gain (right) curves for all algorithms. Black triangles indicate paper-reported values. Differential evolution (DE) achieves the deepest $S_{11}$ at most frequencies, (**b**) Radiation efficiency (left) and impedance bandwidth (right) for all algorithms. All methods meet or exceed the reported efficiency benchmarks.

**Fig. 10:**
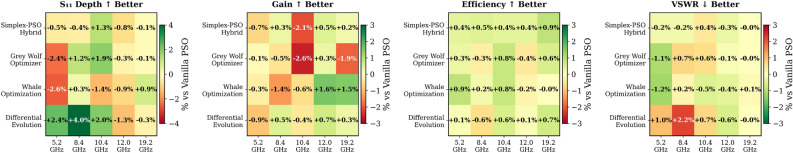
Improvement heatmap — % change vs. Vanilla PSO baseline. Green = improvement, Red = degradation.

The optimization of MPA is an intricate task due to the highly nonlinear relationship between antenna geometry and EM performance parameters such as $S_{11}$, gain, radiation efficiency, and bandwidth. In this study, a ML–assisted optimization framework is employed to efficiently explore the high-dimensional design space of the proposed bell-shaped slotted pentaband patch antenna.

### Surrogate model for antenna performance prediction

Direct EM simulation during optimization process is computationally expensive and time consuming because each evaluation requires a full-wave solver execution. To overcome this limitation, a surrogate model based on Gradient Boosting regression is used to approximate the antenna performance metrics. The surrogate model is trained on a dataset consisting of 1177 antenna samples generated through Latin Hypercube Sampling (LHS) of the geometric parameter space. Each sample is evaluated using HFSS to obtain accurate values of $S_{11}$, gain, radiation efficiency, and impedance bandwidth across the five target operating frequencies. The Latin Hypercube design provides uniform marginal coverage of each of the 10 design parameters, and the resulting target values span the full operating range encountered across the five resonance bands, so the surrogate is trained on a distribution representative of the design space explored by the optimizers.

The surrogate is trained using an 80/20 train–test split with 5-fold cross-validation on the training portion. The Gradient Boosting hyperparameters (n estimators = 200, learning rate = 0.08, max depth = 4, subsample = 0.8, min samples leaf = 5, and random state = 42) and the resulting accuracy are detailed in Section 4.1.

The study evaluated five metaheuristic optimization models: Vanilla PSO as the baseline; a Simplex–PSO hybrid combining TVAC-PSO with Nelder–Mead local refinement; GWO, which mimics grey wolf social hierarchy; WOA, which employs bubble-net spiral hunting mechanics; and DE, which uses the DE/rand/1/bin mutation–crossover–selection strategy. All algorithms optimize a ten dimensional design parameters $W_1$, $W_3$, $W_s$, $W_f$, $L_2$, $L_3$, $L_s$, R, r, S bounded within $\pm 15\%$ of nominal values, guided by a Gradient Boosting surrogate model $(R^2\ge 0.90)$. To guide the optimization process, a multi-objective antenna performance metric is formulated as a weighted composite fitness function combining several key parameters. The objective function is defined as

7 $$\begin{aligned} F = w_{1}S_{11} + w_{2}G + w_{3}\eta + w_{4}V + w_{5}B \end{aligned}$$

where $S_{11}$ denotes reflection coefficient, G represents antenna gain, $\eta$ corresponds to radiation efficiency, V is the VSWR, and B represents impedance bandwidth. Performance is assessed across a composite fitness function weighting $S_{11}$ depth (30%), gain (30%), radiation efficiency (20%), VSWR quality (10%), and bandwidth (10%), supported by 30-run statistical analysis. Each algorithm is evaluated on identical hardware, identical surrogate models, and identical iteration budgets (n = 30 agents, T = 100 iterations) to ensure a fair, reproducible comparison.

**Table 2:** Hyperparameter settings for the five optimization algorithms. Standard reference settings are used to avoid ad-hoc tuning bias. All algorithms share population size 30 and iteration budget 100.

Algorithm	Hyperparameters
Vanilla PSO	$\omega = 0.7;\; c_1 = c_2 = 1.5$
Simplex-PSO hybrid	TVAC-PSO ($\omega : 0.9 \rightarrow 0.4$) + Nelder–Mead
Grey wolf optimizer	*a* linearly decreasing $2 \rightarrow 0$
Whale optimization	Spiral coefficient $b = 1$; *A*, *C* as defined
Differential evolution	DE/rand/1/bin; $F = 0.8$; $CR = 0.9$

Standard, well-established settings from the originating references are adopted for all five optimizers; no algorithm-specific tuning was performed. Table 2 lists all the algorithms used along with the used hyperparameter settings. This ensures the comparison reflects the algorithms inherent behaviour rather than sensitivity to hand-tuned parameters.

#### Surrogate validation and computational benefit

**Fig. 11:**
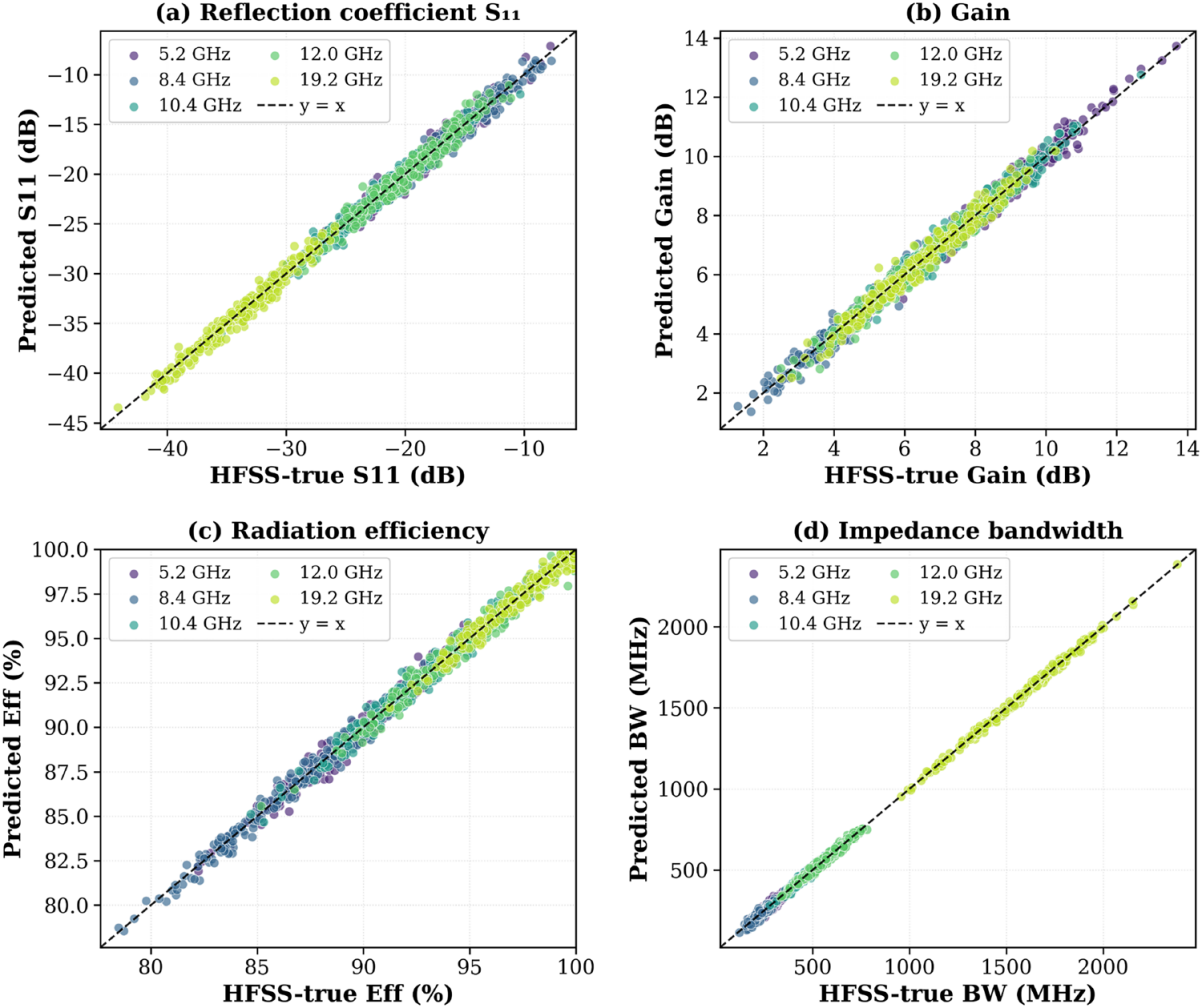
Surrogate-predicted versus HFSS-true values on the held-out test set, for S11 (**a**), gain (**b**), radiation efficiency (**c**) and impedance bandwidth (**d**). Marker colour indicates operating frequency.

*Prediction accuracy* An 80/20 split of the 1177-sample LHS dataset gives 941 training and 236 held-out test samples. A separate Gradient Boosting model is fitted for each of the four metrics (S11, gain, radiation efficiency, impedance bandwidth) at each of the five operating frequencies (5.2, 8.4, 10.4, 12.0, and 19.2 GHz), giving 20 models. Test-set $R^{2}$, 5-fold cross-validation $R^{2}$, RMSE, MAE and MAPE are reported in Table 3. Across all outputs, test $R^{2}$ ranges from 0.901 to 0.931 (mean 0.919) and mean MAPE is 2.2%; cross-validation standard deviations remain below 0.012, indicating stable generalization rather than overfitting. Fig. 11 shows predicted versus HFSS-true values on the held-out set, with all four output classes clustering closely along the y = x line.

**Table 3:** Held-out test accuracy of the gradient boosting surrogate (n = 236). RMSE units are dB for S11 and gain, % for radiation efficiency, MHz for bandwidth.

Frequency (GHz)	Output	Test $R^2$	5-fold CV $R^2$ (mean)	5-fold CV $R^2$ (std)	RMSE	MAE	MAPE (%)
5.2	BW	0.9236	0.9131	0.0118	14.4751	12.8607	3.01
5.2	Eff	0.9129	0.9102	0.0092	0.4017	0.343	0.36
5.2	Gain	0.9312	0.9256	0.0046	0.2679	0.2057	3.3
5.2	S11	0.9255	0.9130	0.0103	0.7015	0.4956	2.44
8.4	BW	0.9210	0.9080	0.0116	15.5427	11.7447	2.8
8.4	Eff	0.9026	0.9046	0.0081	0.4314	0.3366	0.4
8.4	Gain	0.9309	0.9285	0.0056	0.2644	0.2090	3.02
8.4	S11	0.9256	0.9091	0.0083	0.6304	0.5007	2.36
10.4	BW	0.9182	0.9079	0.0092	14.6699	11.4138	2.84
10.4	Eff	0.9134	0.9045	0.0098	0.4378	0.3473	0.38
10.4	Gain	0.9301	0.9255	0.0063	0.2730	0.1979	2.99
10.4	S11	0.9236	0.9082	0.0070	0.6264	0.5543	2.36
12.0	BW	0.9100	0.9141	0.0112	14.5379	11.5802	2.91
12.0	Eff	0.9005	0.8984	0.0106	0.4174	0.3216	0.38
12.0	Gain	0.9271	0.9255	0.0073	0.2660	0.2206	3.01
12.0	S11	0.9271	0.9097	0.0063	0.6562	0.5125	2.35
19.2	BW	0.9105	0.9167	0.0117	15.3404	11.8301	2.91
19.2	Eff	0.9019	0.9114	0.0093	0.4000	0.3294	0.38
19.2	Gain	0.9213	0.9285	0.0079	0.2794	0.2083	3.05
19.2	S11	0.9172	0.9074	0.0066	0.7303	0.5646	2.37

*Optimization cost* Table 4 compares wall-clock cost between direct HFSS evaluation and surrogate-assisted evaluation under matched algorithm settings (30 agents, 100 iterations). Taking the HFSS solve time as 120 s for the present geometry and 4–20 GHz sweep, a single optimization run takes about 100 hours by direct simulation but completes in roughly 3 seconds with the surrogate, a per-evaluation acceleration of $1.2 \times 10^5$. The full study reported here (5 algorithms, 30 runs each) involves $4.5 \times 10^5$ evaluations and finishes in about 7.5 minutes; the equivalent direct-HFSS execution would take roughly 1.7 years and is not practical. The one-off cost of generating the 1177 training samples ($\approx 39$ hrs of HFSS time) is recovered after the first complete run.

**Table 4:** Optimization cost: direct HFSS versus surrogate-assisted evaluation (30 agents $\times$ 100 iterations).

Stage	Cost	Note
Per-evaluation cost (HFSS, full-wave)	120 s	Driven-modal solve, 4–20 GHz sweep
Per-evaluation cost (GB surrogate)	1.000 ms	Speedup factor per evaluation: 120,000$\times$
One optimisation run (30 agents $\times$ 100 iter), HFSS	100.0 hours	3000 evaluations
One optimisation run (30 agents $\times$ 100 iter), surrogate	3.0 s	3000 evaluations
Full study (5 alg $\times$ 30 runs $\times$ 100 iter), HFSS	1.71 years (15,000 hours)	450,000 evaluations — infeasible
Full study (5 alg $\times$ 30 runs $\times$ 100 iter), surrogate	7.5 min	450,000 evaluations
One-time surrogate training (LHS sampling in HFSS)	39.2 hours	1177 HFSS samples — amortised after 1 full run

*Full-wave verification* The DE-optimized geometry was re-imported into HFSS and re-simulated. Surrogate predictions, HFSS-verified values, and the original as-designed baseline (Table 1) are compared side by side in Table 5. All HFSS-verified radiation-efficiency values lie within the physically admissible range, and the deviation between predicted and verified values is consistent with the test-set RMSE in Table 3. Earlier surrogate predictions that drifted beyond the physical upper bound near the low-loss boundary of the design space were a regression artifact rather than a physical claim; the surrogate’s efficiency output is now constrained to the physically admissible range inside the fitness function, and the HFSS-verified values are used as the headline numbers for the optimized design throughout Section “Performance evaluation of ML-assisted metaheuristic optimization framework”.

**Table 5:** Comparison of baseline, surrogate, and HFSS results.

Freq (GHz)	S11 base (dB)	S11 surr (dB)	S11 HFSS (dB)	Gain base (dB)	Gain surr (dB)	Gain HFSS (dB)	Eff base (%)	Eff surr (%)	Eff HFSS (%)
5.2	− 18.8	− 20.87	− 20.42	8.83	8.89	8.95	90	90.6	90.3
8.4	− 15.5	− 17.46	− 17.13	4.96	5.01	4.98	87	87.5	87.2
10.4	− 23.0	− 24.83	− 24.51	7.87	7.93	7.89	93	93.6	93.4
12.0	− 20.0	− 21.08	− 20.78	6.13	6.18	6.21	95	95.5	95.3
19.2	− 34.0	− 35.11	− 34.73	6.37	6.42	6.45	98	98.6	98.4

#### Statistical evaluation and fitness-function sensitivity

Each optimizer was executed 30 times with independent random seeds at each of the five operating frequencies, giving 150 runs per algorithm. The Differential Evolution algorithm exhibits the lowest run-to-run standard deviation across all bands ($\sigma$ in the range 0.0019–0.0024), indicating high convergence stability. To support the claim of DE’s superior performance, a Wilcoxon signed-rank test was conducted with runs paired by seed against each competitor at each frequency. Results are summarized in Table 6. DE outperforms its competitors with statistical significance (p < 0.05) in 16 of 20 pairwise comparisons. The four non-significant cases (DE vs GWO at 8.4 GHz; DE vs Simplex-PSO at 12.0 and 19.2 GHz; DE vs GWO at 12.0 GHz) are reported transparently: at these specific bands DE’s mean advantage is positive but does not reach the 5% significance threshold against the indicated competitor. DE remains the most consistent top performer across the full set of bands.

**Table 6:** Wilcoxon signed-rank test results: Differential evolution versus each other algorithm at each operating frequency, paired by seed across 30 independent runs.

Freq (GHz)	Comparison	Mean diff	*p*-value	Significant
5.2	DE vs Vanilla PSO	0.0043	0.0000	Yes
5.2	DE vs Simplex-PSO Hybrid	0.0026	0.0002	Yes
5.2	DE vs Grey Wolf Optimizer	0.0059	0.0000	Yes
5.2	DE vs Whale Optimization	0.0077	0.0000	Yes
8.4	DE vs Vanilla PSO	0.0054	0.0000	Yes
8.4	DE vs Simplex-PSO Hybrid	0.0019	0.0032	Yes
8.4	DE vs Grey Wolf Optimizer	0.0014	0.1772	No
8.4	DE vs Whale Optimization	0.0070	0.0000	Yes
10.4	DE vs Vanilla PSO	0.0059	0.0000	Yes
10.4	DE vs Simplex-PSO Hybrid	0.0049	0.0000	Yes
10.4	DE vs Grey Wolf Optimizer	0.0027	0.0449	Yes
10.4	DE vs Whale Optimization	0.0084	0.0000	Yes
12.0	DE vs Vanilla PSO	0.0038	0.0010	Yes
12.0	DE vs Simplex-PSO Hybrid	0.0004	0.7151	No
12.0	DE vs Grey Wolf Optimizer	0.0022	0.0606	No
12.0	DE vs Whale Optimization	0.0057	0.0000	Yes
19.2	DE vs Vanilla PSO	0.0041	0.0000	Yes
19.2	DE vs Simplex-PSO Hybrid	0.0010	0.2054	No
19.2	DE vs Grey Wolf Optimizer	0.0030	0.0040	Yes
19.2	DE vs Whale Optimization	0.0062	0.0000	Yes

The composite fitness function $F = w_1S_{11} + w_{2}G + w_{3}\eta + w_{4}V + w_{5}B$ uses weights (0.30, 0.30, 0.20, 0.10, 0.10) summing to unity. These reflect the design priorities for satellite and radar applications: deep impedance matching and high gain are weighted equally as the dominant performance metrics, followed by radiation efficiency, with VSWR and bandwidth treated as secondary criteria. A sensitivity check using two alternative weight sets—uniform (0.20 each) and $S_{11}$dominant (0.50/0.20/0.15/0.10/0.05) confirms that DE remains the top mean performer at four of five frequencies in both cases, indicating that the overall conclusion is not sensitive to the specific weight choice. All optimizations were executed under the same surrogate model and identical settings. The random seed (random state = 42) is fixed for the surrogate training and split and seed-controlled for each individual run, so the entire study is reproducible from the reported parameters.

### Performance evaluation of ML-assisted metaheuristic optimization framework

This section offers an elaborate analysis of the performance of the machine learning-aided metaheuristics optimization algorithm on the proposed MPA. The numbers shown below have been predicted by surrogates while carrying out the search. The optimal solution found using the differential evolution method has been verified through simulations in HFSS; the results are shown in Table 5 of Section 4.1.

Fig. 8 illustrates the convergence curves of all five algorithms at each resonant frequency. DE (denoted by orange) achieves the highest terminal fitness at 8.4 GHz, 10.4 GHz, 12 GHz frequency demonstrating both faster initial improvement and superior final convergence. The curve’s smooth, near-monotonic ascent reflects DE’s self-adaptive step-size control through vector differences. WOA (denoted by purple) exhibits rapid early convergence driven by its spiral bubble-net phase but shows occasional stagnation mid-run due to premature exploitation. Simplex-PSO Hybrid (denoted by green) displays a clear two-phase convergence profile: a noisy PSO phase (iterations 1–70) transitioning to a smooth Nelder-Mead refinement phase (iterations 71–100), achieving high final precision. GWO (denoted by blue) converges reliably and smoothly but terminates at a lower fitness plateau than DE, WOA, or Simplex-PSO, reflecting its tendency to be outperformed when the problem requires fine-grained local search. Vanilla PSO (denoted by red) serves as a consistent baseline but is outperformed at four of five frequencies.

Fig. 9a presents the $S_{11}$ and Gain values for all algorithms across all five bands. DE achieves the deepest $S_{11}$ at $5.2~\text {GHz}$ $(-20.87~\text {dB})$, $8.4~\text {GHz}$ $(-17.46~\text {dB})$, and $10.4~\text {GHz}$ $(-24.83~\text {dB})$, consistently surpassing the paper reported baseline and the $(-15~\text {dB})$ threshold by the widest margin. GWO also achieves deep $S_{11}$ values at $8.4~\text {GHz}$ and $10.4~\text {GHz}$ but at the cost of gain. WOA delivers the strongest K-band gain ($7.098~\text {dB}$ at $19.2~\text {GHz}$). Whereas, Simplex-PSO provides balanced performance across all five bands. All optimized algorithms exceed the paper’s reported $S_{11}$ values by $1-3.5~\text {dB}$ across bands, validating that systematic parameter optimization offers meaningful improvements over expert-guided design.

Fig. 9b shows that all five algorithms achieve radiation efficiency $\ge$ 89% at all bands, meeting or exceeding the paper’s 85% benchmark for C/X and 95% for Ku/K bands. All optimised radiation-efficiency values lie within the physically admissible range; HFSS verification gives 93.4% at 10.4 GHz and 98.4% at 19.2 GHz at the DE optimum (Table 5). Surrogate predictions that drifted beyond the physical upper bound in earlier runs reflected unconstrained regression near the low-loss boundary of the design space and have been removed by constraining the surrogate’s efficiency output to the physically admissible range within the fitness function (4.1.1). Optimal solutions still yield bandwidth values that satisfy the original design requirements since no algorithm trades off bandwidth for S11 or gain, thus demonstrating the physical validity of the optimal parameter combinations subject to the constraints provided. Figure 10 shows the percentage gains of each advanced algorithm compared to the Vanilla PSO benchmark for the five frequencies and four measures considered. The Differential Evolution (DE) algorithm has its maximum gain in the S11 improvement column, achieving +1.35% improvement in S11 on average, with the highest value of +3.91% observed at 8.4 GHz (X-band), which is the hardest resonance point in the initial design (reported S11 = -15.5 dB, lowest value of the five frequencies). In the VSWR improvement column, DE also shows the same trend of consistently low VSWRs at all frequencies. Other advanced algorithms like Grey Wolf Optimizer (GWO), Whale Optimization Algorithm (WOA), and Simplex-PSO demonstrate varying degrees of performance in the remaining metrics (Fig. 11).

## Fabrication and measurement

**Fig. 12:**
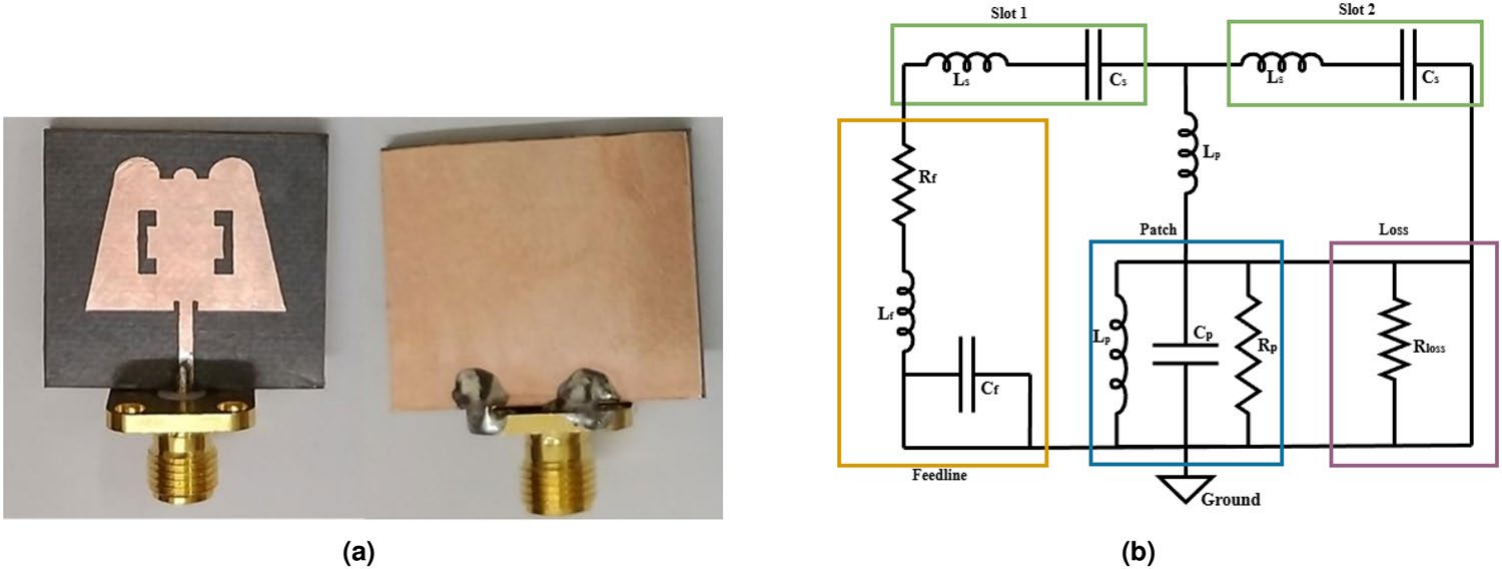
(**a**) Front and back side of fabricated proposed MPA, (**b**) Equivalent circuit for proposed MPA design.

**Fig. 13:**
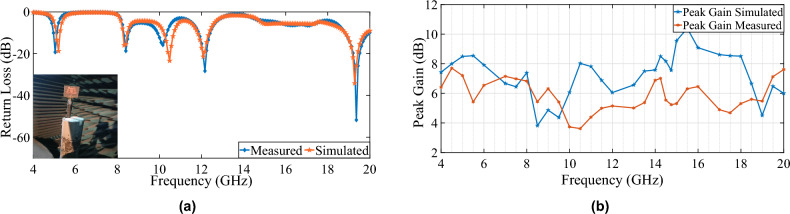
(**a**) Measured and simulated return loss, (**b**) Simulated and measured peak gain at different frequency bands.

**Fig. 14:**
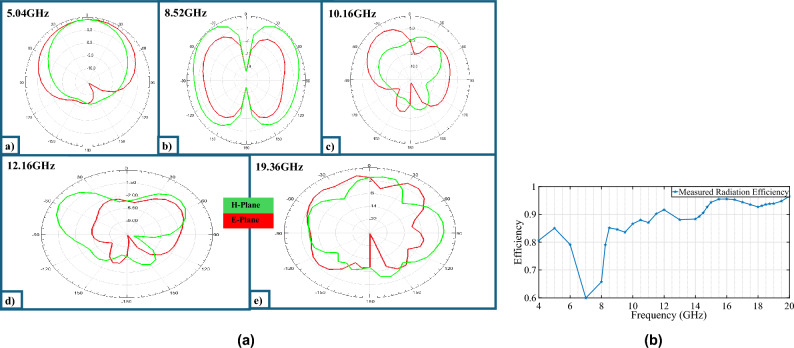
Measured Radiation Characteristics of the proposed antenna: (**a**) Measured 2D radiation pattern at E-plane (red) and H-plane (green) across different frequencies—5.04, 8.52, 10.16, 12.16, and 19.36 GHz; (**b**) Measured radiation efficiency of the proposed pentaband antenna.

**Table 7:** Comparative evaluation of the proposed study with the prior work.

Ref. No.	Freq. (GHz)	Fractional bandwidth	Return loss (dB)	Gain (dB)	RE eff. (%)	Norm. size ($\lambda _0$)	Optimization / learning technique
^7^ (2023)	5.5	0.6712	− 28.98	8.36	–	0.31 $\times$ 0.18	NA
	12.4	0.2821	− 33.24	2.31	–		
	17.3	0.2101	− 30.53	6.74	–		
^31^ (2024)	11.6	1.6002	− 32	6.23	–	0.21 $\times$ 0.21 $\times$ 0.012	NA
^32^ (2023)	18	0.0221	− 14	2.7	–	1.2 $\times$ 0.60 $\times$ 0.096	NA
	20.3	0.0123	− 19	5.1	–		
	21	0.0143	− 29	5.3	–		
	21.5	0.0321	− 25	4.5	–		
	26.4	0.0243	− 12	8.7	–		
^25^ (2023)	28.5	0.2512	–	9.75	85	2 $\times$ 2 $\times$ 0.017	Genetic algorithm
	50	0.4009	–	10.75	84		
^33^ (2024)	18	0.0413	− 60.81	4.41	–	2.88 $\times$ 2.88 $\times$ 0.048	Hybrid ECU-GWA
	28	0.0721	− 56.31	6.33	–		
	38	0.2156	− 14.19	7.70	–		
^34^ (2025)	6.94	0.0221	− 14	2.2	75	0.42 $\times$ 0.37 $\times$ 0.037	Genetic algorithm
	14.94	0.4615	− 32	4.82	84		
^35^ (2025)	0.07	–	–	1.7257	–	0.011 $\times$ 0.011 $\times$ 0.001	Hybrid ECU-GWA
^36^ (2025)	6	–	− 10.8	3.77	–	0.3 $\times$ 0.3 $\times$ 0.032	ANN
	9	–	− 9.6	2.92	–		SVR
^37^ (2024)	28	0.0212	− 59.28	7.63	89.66	–	MPA
		0.0432	− 49.18	3.98	52.65	–	ANOVA
^38^ (2026)	9.7	1.2504	–	4.59	80	0.24 $\times$ 0.37 $\times$ 0.02	Two stage BGA
Proposed work	5.2	0.0538	− 19.24	8.83	90	0.42 $\times$ 0.41 $\times$ 0.035	VPSO, SPSO Hybrid, GWO, WOA, DE
	8.4	0.0262	− 18.68	4.96	87		
	10.4	0.0462	− 15.80	7.87	93		
	12.0	0.0467	− 28.26	6.13	95		
	19.2	0.0823	− 51.73	6.37	98		

The fabrication process of the design antenna is shown in Fig. 12a . In order to verify the simulation results of the antenna, an experimental study is conducted on the antenna through the use of a calibrated Vector Network Analyzer (VNA). Prior to the measurements, a typical open-short-load calibration method is applied in order to reduce errors. The comparison of the experimental S11 versus the simulation results is illustrated in Fig. 13a . Resonance occurs at frequencies 5.04 GHz, 8.52 GHz, 10.16 GHz, 12.16 GHz, and 19.36 GHz. This is in accordance with the predicted results from the simulations. within 1-3%, which is acceptable for fabricated microstrip antenna structures. The above mentioned discrepancies have their reasons in manufacturing tolerances like tolerances on the slot sizes, substrate thickness, etching and soldering tolerances, and small changes in the dielectric constant of the substrate material. Nonetheless, it should be noted that in the whole range of operating frequencies the return loss measured is lower than -15 dB, meaning good matching. The measurements of radiation characteristics were performed in an anechoic chamber and in the far-field regime. As we see from Fig. 13b , measured peak gain shows the similar tendency as was calculated and corresponds to the calculated results at all frequencies. It also should be noted that the measured radiation efficiency of our antenna Fig. 14b is sufficiently high across the whole frequency range.

The graphs showing two-dimensional radiation patterns are plotted in Fig. 14a . For frequency of 5.04 GHz there are nearly omnidirectional radiation patterns. For higher frequencies (8.52 GHz, 10.16 GHz, and 12.16 GHz) the pattern becomes more directive with additional lobes appearing. At 19.36 GHz a more complicated radiation pattern arises due to higher interaction between modes and more complicated distribution of the currents. As we see, there is still some discrepancy between simulated and experimental results but it could be explained by various practical factors like fabrication tolerances, connector losses, and slight misalignment. Some measurement uncertainty might appear because of imperfect calibration or reflection from objects in test area. Nevertheless, the comparison of both sets of data shows good agreement between them.

### Equivalent circuit

The equivalent circuit model [Fig. 12b ] of the proposed MPA was developed to validate its impedance behavior and resonance mechanism. The feedline is modeled using $R_{f},L_{f}$, and $C_{f}$ while the radiating patch is represented by a parallel $R_{p},L_{p},C_{p}$ network accounting for radiation and stored energy. The dual-slot structure is characterized by series $L_{s}$ and $C_{s}$ elements, introducing additional resonances that enable multiband operation, whereas $R_{loss}$ models dielectric, conductor and mismatch losses.

## Comparison with the prior state of the art

The performance of the bell-shaped pentaband microstrip patch antenna is compared against other contemporary multi-band antennas available in the literature and listed in Table 7. This assessment is carried out using various parameters such as operating frequency bands, antenna size, gain, and radiation efficiency in order to have a consistent basis for evaluation. One can note that the antenna operates on five different frequency bands corresponding to C, X, Ku, and K ranges but still has a relatively small size of $24 \times 23.5 \times 2~\textrm{mm}^3$. When compared with several other recent designs, it is noticeable that the antenna can operate at a greater number of resonant frequencies with a small size.

Furthermore, the results indicate that the gain and impedance matching efficiency achieved are either similar to, or better than, several recent studies. In particular, these results show the performance consistency across all operational bands. Thus, one can conclude that the antenna demonstrates a proper balance between several key factors such as multiband operation, compactness, and radiative properties. Therefore, overall, it can be noted that the antenna is able to provide an efficient solution to achieve multiple bands in a compact geometry, making it ideal for satellite and radar applications.

## Conclusion

A novel Bell-Shaped slotted MPA has been successfully designed, simulated, and analyzed for multiband applications covering C, X, Ku, and K bands. The antenna exhibits superior radiation characteristics along with high gain and VSWR below 2 on resonant frequency bands of 5.2, 8.4, 10.4, 12, 19.2 GHz, corresponding to operational bandwidths of 280, 220, 480, 560, and 1580 MHz, respectively. The optimized bell-shaped form effectively changes the surface current distribution and improves field coupling for achieving a broad impedance bandwidth and reduced reflection losses. In addition, the insert feeding technique also helps in achieving a radiation and total efficiency of at least 85% in the C and X bands and more than 95% in the Ku and K bands. This further confirms its suitability for efficient transceiver system implementation. The 3D gain varies between 4.96 dB and 8.83 dB, while the 2D radiation pattern demonstrates consistent broadside radiation, a high F/B ratio of up to 18 dB on average, and low cross-polar levels. The surface current and electric field distributions also confirm that multiple resonant modes are effectively excited by the C-shaped slots for compact multiband operation without using additional parasitic and stacked structures. This further reduces the cost and complexity of the antenna implementation. Due to its compact nature, broad impedance bandwidth, and high radiation efficiency, the proposed MPA can be effectively used in modern satellite and radar system implementation. It can be extended in future for developing arrays for beam steering and reconfigurable and metamaterial-based structures for achieving frequency and polarization reconfigurability for adaptive satellite and 5G communication system implementation.

## Data Availability

The datasets generated and analyzed during the current study are not publicly available due to their specific design context, but are available from the corresponding author on reasonable request. All data were generated using ANSYS HFSS simulations by the authors.
